# Gels as Promising Delivery Systems: Physicochemical Property Characterization and Recent Applications

**DOI:** 10.3390/pharmaceutics17020249

**Published:** 2025-02-14

**Authors:** Enzhao Wang, Zhaoying Qi, Yuzhou Cao, Ruixiang Li, Jing Wu, Rongshuang Tang, Yi Gao, Ruofei Du, Minchen Liu

**Affiliations:** 1 Innovation Research Institute of Traditional Chinese Medicine, Shanghai University of Traditional Chinese Medicine, Shanghai 201203, China; 22022622@shutcm.edu.cn (E.W.); 22023645@shutcm.edu.cn (Z.Q.); ruixiangli@shutcm.edu.cn (R.L.); 22024754@shutcm.edu.cn (R.T.); 2 Engineering Research Center of Modern Preparation Technology of TCM of Ministry of Education, Shanghai University of Traditional Chinese Medicine, Shanghai 201203, China; 3 School of Science, National University of Singapore, Singapore 119077, Singapore; yuzhou2023go@163.com; 4 School of Pharmacy, Zhejiang Pharmaceutical University, Ningbo 315100, China; wujingzjpc@126.com; 5 School of Pharmacy, Shanghai University of Traditional Chinese Medicine, Shanghai 201203, China; a13701656307@163.com

**Keywords:** gels, physicochemical property characterization, organogel, hydrogel, rheological property, gel structure

## Abstract

Gels constitute a versatile class of materials with considerable potential for applications in both technical and medical domains. Physicochemical property characterization is a critical evaluation method for gels. Common characterization techniques include pH measurement, structural analysis, mechanical property assessment, rheological analysis, and phase transition studies, among others. While numerous research articles report characterization results, few reviews comprehensively summarize the appropriate numerical ranges for these properties. This lack of standardization complicates harmonized evaluation methods and hinders direct comparisons between different gels. To address this gap, it is essential to systematically investigate characterization methods and analyze data from the extensive body of literature on gels. In this review, we provide a comprehensive summary of general characterization methods and present a detailed analysis of gel characterization data to support future research and promote standardized evaluation protocols.

## 1. Introduction

Gels are three-dimensional networks formed by colloidal particles in combination with aqueous, non-aqueous, or hydroalcoholic materials. This semi-solid preparation is characterized by its spreading ability and notable cooling effect, due to its humectant properties [1,2]. Gels have become an integral part of daily life, with widespread applications in products such as soaps, shampoos, and toothpastes [3].

The term ‘gel’ was coined by Thomas Graham, derived from ‘gelatine’, which originates from the Latin noun “gelatum”, meaning a congealed liquid [4]. Research on gels began over a century ago [5], and both hydrogels and organogels have witnessed significant advancements in pharmaceutical applications during this time. The origins of hydrogels date back to 1894, when the term ‘hydrogel’ was first mentioned in the literature. In 1960, Wichterle and Lim published a landmark paper that laid the foundation for modern hydrogel research, particularly in biomedical applications [6,7]. Subsequently, Hui and Robinson reported the first bioadhesive ocular drug delivery system using hydrogels in 1985, which improved the bioavailability of therapeutic drugs [8]. Hydrogels also gained importance in wound care, as demonstrated by the 1994 patent for hydrogels in wound dressings by Cartmell and Sturtevant [9].

The innovation continued with the development of DNA-containing polymer hydrogels by Nagahara and Matsuda in 1996, adding programmable features to hydrophilic materials [10]. In 1997, poly(ethylene glycol)-based (PEG-based) injectable hydrogels were introduced as the first biodegradable thermosensitive hydrogels, revealing their potential as injectable biomaterial [11]. Superporous hydrogels (SPHs), introduced in 1999, represented a new generation of hydrogels with enhanced water absorption capabilities [12]. The first lipid nanoparticles (LNP)–hydrogel composite for transdermal drug delivery of lipophilic drugs was developed in 2004 [13].

Organogels, on the other hand, have a shorter history, beginning in 1988, when Scartazzini and Luisi first utilized lecithin to design organogels [14]. Since then, organogels have been explored for various administration routes, including their use as matrix systems for transdermal drug transport in 1991 [15], oral delivery in 2005 [14], and transnasal absorption in 2004 [16]. In the same year, L-alanine derivatives were used to develop injectable organogels with excellent biocompatibility and biodegradability [17].

Today, the development of novel smart gels continues to accelerate with advancements in modern technology, paving the way for innovative drug delivery systems. The historical timeline of gel development is illustrated in Figure 1.

Gels represent a versatile category of substances with significant potential for technical and medical applications. Based on the type of liquid immobilized within their three-dimensional structure, gels are broadly classified into two categories: hydrogels and organogels [18].

Hydrogels, characterized by their water-attracting three-dimensional network capable of absorbing substantial amounts of water or biological fluids, are widely regarded as promising materials for biomedical applications [18]. Recent research has extensively explored their potential in various fields, including tissue engineering [19,20], drug delivery [21], wearable electronics [22], and wound dressings [23,24,25]. In contrast, the lower degree of hydration, potential toxicity, and solvent usage of organogels have limited their applications [26,27]. However, considerable efforts have been made to enhance their suitability for drug delivery [28], with applications in ocular drug release [29], transdermal delivery [30], and intravaginal delivery [31].

The properties of gels are intrinsically linked to their structure, which, in turn, determines their functionality. Accurate and effective applications necessitate a thorough understanding of gel properties, adhering to the principle of a ‘case-by-case’ approach. Consequently, the characterization of gel properties is crucial for their study across fields such as materials science, pharmaceuticals, and wearable electronics [32,33,34]. With advancements in scientific research, gels have undergone extensive characterization using a variety of techniques and methods to enhance their functionality [27].

For instance, pH measurements enable the optimization of drug compatibility with specific administration routes. Microscopy techniques, such as scanning electron microscopy (SEM) and transmission electron microscopy (TEM), reveal the three-dimensional microstructure and morphology of gels [35,36]. Spectroscopy methods, including nuclear magnetic resonance (NMR), circular dichroism (CD), and Fourier transform infrared (FT-IR), facilitate the analysis of molecular interactions and internal dynamics [37,38]. Diffraction techniques, such as small-angle X-ray scattering (SAXS) and X-ray diffraction (XRD), provide insights into gel fiber size, packing patterns, and crystalline structure [39,40]. Mechanical properties, including gel strength and cohesiveness, can be assessed using a texture analyzer [41]. Rheological studies evaluate the dynamic shear modulus, viscoelastic behavior, thermal stability, sol-to-gel transition temperatures, viscosity (η), storage modulus (G′), and loss modulus (G″) [42,43].

In this review, we systematically analyze the application of these characterization techniques through five specific analyses, as illustrated in Figure 2. Despite the extensive research on gels, no comprehensive review has summarized the characterization methods across the field of gel materials. Addressing this gap, we present a detailed review of characterization methods for gels in biomedical applications, focusing on physical characterization. This review provides a global perspective on gels, encompassing their definition, development, properties, characterization methods, and applications. It aims to establish a foundation for future research and development of gel materials and to offer strategic guidance for designing and characterizing gels in various applications.

## 2. Physical Properties of Gels

Gels, as versatile materials, can be applied to various body regions, including the skin [44], oral cavity [45], and bone tissue [46]. Each application imposes distinct requirements on the physical properties of gels, necessitating thorough characterization to ensure their structural integrity, stability, and safety. This process is essential for the development of reliable and effective formulations. In this review, we present an overview of five key physical properties of gels, highlighting their importance in diverse biomedical applications.

### 2.1. pH

pH is a critical parameter in the quality evaluation of gels, as it directly impacts the effectiveness and safety of the encapsulated drugs, as well as patient comfort during administration. The required pH value varies across different types of gels, such as transdermal gels, injectable gels, and ophthalmic gels, depending on their specific pharmaceutical applications and routes of administration [47].

The safety and biocompatibility of gels are critical considerations with respect to their pH, as pH directly influences drug release, swelling behavior, and structural transformations. Notably, smart-responsive gels, such as pH-sensitive gels, have garnered significant research attention in recent years due to their tunable properties.

Beyond pH, ionic strength, although less commonly characterized, plays a crucial role in determining gel properties and applications. Low ionic strength enhances charge repulsion, leading to increased swelling and a more relaxed gel structure. Conversely, high ionic strength neutralizes charged groups, reduces repulsive forces, and diminishes gel swelling [48]. An ionic strength approximating physiological conditions is optimal, as it modulates the swelling degree [49], water uptake [48], drug release [50], and other functional properties.

### 2.2. Structure

Understanding the nanoscale and microscale structural composition of polymer networks is essential for determining the macroscopic properties of gels [51]. The equilibrium between hydrophobic and hydrophilic states, solvent characteristics, and gelator–solvent interactions, including diverse noncovalent forces, such as hydrogen bonding, ionic interactions, π–π stacking, and van der Waals forces, contribute to the formation of supramolecular gels. These gels exhibit varied morphologies depending on the gelator, solvent, and gelation conditions [52]. Comprehensive structural analysis at the supramolecular scale is critical to fully characterize gel systems.

Advanced imaging techniques, particularly electron microscopy, are widely employed to analyze the structural properties of gel. Microscopy enables detailed observation of gel morphology at nanometer and micrometer scales, providing critical insights into their structural organization. Among these techniques, SEM is the most commonly utilized [53]. SEM is typically used to evaluate pore formation, pore dimensions, crosslinking conditions, and the effects of specific loading substances on the overall gel structure [52,54,55,56,57]. The integration of microscopic methods offers a comprehensive understanding of the intricate configurations of gel networks [35,58].

Both SEM and TEM provide valuable insights into the micro- and nanostructures of gels, offering detailed visualization of the morphology of aggregates formed during gelation [59]. Cryo-scanning electron microscopy (cryo-SEM), an emerging technique for structural characterization, addresses the challenge of directly observing the 3D structure of liquid, semi-liquid, and hydrated samples. Cryo-SEM has gained significant attention in biomedical applications for its ability to visualize liquid or uncrystallized proteins, which are challenging to analyze using conventional SEM techniques [60,61]. Additionally, cryo-SEM provides the advantage of maintaining the gel’s internal environment at low temperatures and under high vacuum, making it particularly useful for examining high-water-content gels or those with internal water that is difficult to remove. This capability enhances the observation of the internal structure of gels, facilitating more accurate structural characterization and advancing their application in various fields.

While electron micrographs provide valuable insights into gel structures, they are most effective when combined with supplementary techniques, such as scattering methods [62]. Small-angle scattering (SAS) techniques, including small-angle neutron scattering (SANS) and small-angle X-ray scattering (SAXS), are particularly useful for analyzing the spatial arrangement of fibrillar networks [63]. Additionally, XRD provides information on crystal types, crystalline structures, and molecular packing within gels [40]. These techniques are ideally suited for examining the skeletal structure of fibrillar networks and the physical properties of gels at the atomic level.

Spectroscopic methods, including NMR, ultraviolet–visible (UV) spectroscopy, fluorescence spectroscopy, circular dichroism (CD), and infrared (IR) spectroscopy, further enhance the understanding of gelation systems [62]. NMR is especially valuable for elucidating molecular structures, material behavior, molecular interactions, and internal dynamics in gels [35,64]. It also enables the measurement of sol-to-gel transition temperatures, corroborating data on thermoreversibility and interactions such as π–π stacking and hydrogen bonding [65]. UV and fluorescence spectroscopy track the absorption and fluorescence properties of gels, while CD evaluates how chiral samples absorb circularly polarized light across wavelengths, offering insights into self-assembly mechanisms, particularly in chiral gelators [37].

Infrared spectroscopy, particularly FT-IR spectroscopy, is extensively used to identify functional groups and assess the extent of hydrogen bonding. It provides detailed information on structural and dynamic changes at the molecular level [66]. Furthermore, FT-IR is an effective method for verifying gel formation and the encapsulation of drugs within the gel matrix [67]. FT-IR is commonly employed to characterize gels, particularly to investigate the interactions between drugs and excipients, including polymers. In such analyses, samples are typically mixed with potassium bromide (KBr) in a defined ratio to form pellets, which are then scanned across a specified wavelength range. Changes in absorbance are monitored to confirm that the incorporation of excipients does not alter the drug’s properties within the gel matrix [68,69,70,71]. For studies focusing on the gel itself, a thin, uniform gel film is often prepared for FT-IR analysis, enabling efficient transmission of infrared light [72]. Although less common, other sample formats, such as solutions or semi-solid forms, may also be utilized for specific applications.

In summary, integrating microscopy with spectroscopy enables a comprehensive understanding of the molecular and supramolecular structures of gels, facilitating advancements in their design and application.

### 2.3. Mechanical Properties

The mechanical properties of gels reflect their ability to resist pressure and deformation [73]. However, the specific mechanical requirements vary across different applications. For instance, hydrogels used in bone tissue engineering must exhibit sufficient mechanical strength to endure the compressive loads inherent to tissue, preventing the collapse of developing structures [74,75]. In wound healing, high pressure resistance is a critical parameter for clinical skin dressings, ensuring durability and functionality [76].

Conversely, for applications such as wearable sensors, the brittleness of hydrogels can hinder their tensile strength and flexibility, limiting their performance. Therefore, achieving a high degree of mechanical robustness is crucial for gels designed for wearable devices [77]. The mechanical properties required for gels across various applications are illustrated in Figure 3 [74,78]. Testing and optimizing these properties are essential for advancing the development and application of gels in diverse fields.

The mechanical properties of gels can be evaluated using various devices, each employing specific testing methods. A dynamic mechanical analyzer (DMA) is commonly used to assess the mechanical characteristics of gels under static and cyclic compression at controlled rates [79]. A texture analyzer measures mechanical robustness by compressing gels at a fixed speed until failure, providing parameters such as Young’s modulus, failure stress, and the number of failure layers [41]. Rheological studies, complemented by sensor-based characterizations, also demonstrate a gel’s resistance to pressure [80]. Additionally, an electronic universal testing machine can evaluate gel stiffness under constant strain at a given temperature while varying the frequencies [81].

The amount of gel sample used for texture analysis testing is determined by different types of texture analyzers and different types of gels. For example, most gel samples are weighed to a specific weight [82,83,84,85]. There are also vials equipped with texture analyzers that determine the sample amount [86,87,88]. Additionally, some methods specify gel dimensions and take samples based on length, width, and height criteria [89,90]. If a standard amount or a standard size of the gel sample measured by the texture analyzer can be established, the error in mechanical strength measurements can be significantly reduced.

The mechanical properties of gels are critical for ensuring formulation efficacy and patient care. Key mechanical attributes include gel strength, spreadability, adhesiveness, and cohesiveness. These properties significantly influence the performance of gel formulations. The physical significance, testing methods, and applications of these properties will be discussed in detail in Section 3.3.

### 2.4. Rheological Property

Rheological parameters are critical quality attributes of gels influencing their manufacturing process, appearance, stability, sensory properties, and in vivo performance. Understanding the rheological properties of gels helps elucidate the relationship between their chemical composition and macroscopic behavior, which is crucial for designing gels with specific characteristics tailored for particular applications [91]. Rheological analysis also provides insights into the structure–property relationships of gels. The primary objective of rheological research is to determine viscoelastic properties, such as η, G′ and G″ [92], and to identify the gel–sol transition temperature. Common rheological tests include strain sweep, frequency sweep, temperature sweep, and creep-recovery tests [35,93]. The working principle of a rheometer during sample testing is illustrated in Figure 4.

Strain sweep tests (amplitude sweeps) are typically conducted at a fixed frequency (usually 1 Hz) at 25 °C, measuring G′ and G″ as the strain increases from 0.01% to 100%. The resulting amplitude sweep curve identifies the linear viscoelastic region (LVR) [94], a plateau where the moduli remain nearly unchanged at low shear strain, with G′ predominating. Beyond the LVR, the G′-G″ crossover point marks the gel–sol transition, where the gel begins to behave like a fluid [91]. The strain value obtained from the LVR serves as the basis for subsequent tests, such as time sweeps, temperature sweeps, and creep-recovery experiments.

Frequency sweep tests are conducted at a fixed shear strain within the LVR to observe the variation of G′ and G″ with frequency, typically ranging from 0.1 to 10 Hz [57,95] or angular frequencies between 0.1 and 100 rad/s. The intersection point of the frequency sweep curve reveals the gel’s cross-linking behavior and the potential presence of a reversible network [91]. These tests also assess the influence of additives on moduli values and the shift of the crossover point.

Temperature sweep tests (temperature ramps) analyze the structural robustness of hydrogels under varying temperature conditions. By gradually increasing the temperature while maintaining a fixed shear strain (within the LVR) and frequency (usually 1 Hz), the viscoelastic parameters are tracked. The temperature at which G′ and G″ intersect represents the gelation temperature, marking the sol-to-gel transition [96,97,98].

Creep tests evaluate the slow deformation of gels under constant stress. Continuous stress is applied to the sample, leading to increasing strain that eventually stabilizes. Upon stress removal, the recovery process is documented over a set duration. The resulting curve, comprising a loading part (creep curve) and a recovery part (creep recovery curve), provides insights into the elastic behavior and stiffness of the gel. The J value (compliance = strain/stress) during loading is inversely proportional to stiffness, while the recovery slope and compliance reflect the gel’s elastic characteristics [99]. Table 1 introduces the main research methods of rheology, image, common rules, and their significance.

The amount of gel used for rheometer testing is mainly determined by the type and size of the fixture on the rheometer, and there is no fixed sample size. In addition, the viscosity of the gel also affects the sample size used for rheological testing to some extent. The types of rheometer fixtures and those generally used for testing will be described in Section 3.4.

### 2.5. Phase Transition

The formation of most gels involves a phase transition from a sol to a gel state [100]. This transition and the stability of the resulting gels are influenced by several factors, including temperature and pH levels. Recently, the development of thermosensitive hydrogels, which undergo sol–gel transitions without requiring chemical reactions, has attracted considerable interest in the biomedical and pharmaceutical fields [101]. A defining characteristic of thermosensitive gels is their temperature responsiveness, with the phase transition temperature playing a crucial role in the shift from sol to gel as the temperature increases [102].

The sol-to-gel transition temperature (Tsol-gel), also known as the phase transition temperature, is a critical parameter for evaluating the thermosensitivity of in situ gels. Understanding this temperature is essential for designing gels tailored to specific applications. This section focuses on the factors influencing gel phase transitions and examines the importance of Tsol-gel in the characterization of thermosensitive gels.

The gel phase transition temperature is defined as the specific temperature at which a gel solution transitions into a gel, commonly identified as the point where the G′ equals the G″ [103]. For thermosensitive gels, this transition temperature largely depends on the properties of the thermosensitive polymers. As the temperature increases, hydrophilic units undergo dehydration, which intensifies hydrophobic interactions among molecular chains [104].

Determining the phase transition temperature is crucial for ensuring gel formation at targeted sites within the human body and for assessing the impact of various additives on the gel’s transition behavior.

As illustrated in Figure 5, various methods are available to measure the phase transition temperature of thermosensitive gels. These include the test tube inversion method and the falling ball method [105]. Additionally, the phase transition temperature can be determined using instruments such as magnetic stirrers and rheometers [106].

The test tube inversion method is the simplest technique for determining the sol-gel transition temperature of a gel. In this method, the sol phase is identified as a flowing liquid when the test tube is inverted, while the gel phase is characterized by a non-flowing state [107]. A water bath, typically within the range of human body temperature, is used to monitor the sol–gel transition at set intervals across different temperature points [108]. The temperature at which the gel ceases to flow is recorded as the transition temperature.

The falling ball method, in addition to measuring the transition temperature, can plot the relationship between temperature and dynamic viscosity. This approach measures the time it takes for a weighted ball to travel a specified distance through the sol or gel phase [109]. The temperature at which dynamic viscosity exhibits a significant change indicates the gel’s transition temperature. Compared to the test tube inversion method, the falling ball method provides more precise and reliable data, with minor differences potentially attributed to the longer duration of the latter technique [110].

The magnetic stirrer method is another simple yet effective way to determine the sol–gel transition temperature. In this approach, a magnetic rod and a digital heat sensor are immersed in the solution, which is heated in a sealed container and gently stirred. When gelation occurs, the magnetic rod ceases to move, and the temperature displayed is recorded as the transition temperature [106,111].

Rotary rheometers are also commonly used to measure the phase transition temperature. This method involves conducting dynamic measurements at a fixed frequency (typically 0.01 to 1 Hz) across a temperature range, including physiological temperatures [106].

The phase transition temperature of thermosensitive gels primarily depends on the lower critical solution temperature (LCST) of the thermosensitive polymers used. The LCST is the temperature below which a polymer can completely dissolve in a solvent to form a homogeneous solution and the temperature above which the polymer undergoes phase separation and the solution becomes turbid. It is primarily measured using a spectrophotometer or a thermostatic cuvette. In drug delivery systems, polymers with LCST properties are utilized to encapsulate drugs. When the temperature rises above the LCST, the polymer undergoes phase separation, and the encapsulated drug can be released at a specific site. For example, some temperature-sensitive hydrogels can be used for localized drug delivery, where a phase transition occurs near body temperature to achieve controlled drug release [112,113]. Moreover, polymers with LCSTs near human body temperature ensure that the gel remains in a sol state at ambient temperature and transitions to a gel state at body temperature [114]. Commonly used temperature-sensitive polymers include synthetic polymers, like N-Isopropylacrylamide copolymers [115,116], polyethylene oxide/polypropylene oxide (PEG/PPO) block copolymers [117,118], and natural polymers, like chitosan [119,120]. These polymers have distinct phase transition mechanisms, which will be discussed in Section 3.5. Additionally, this section will elaborate on the effects of various additives on Tsol-gel, highlighting their advantages, limitations, and potential applications.

## 3. The Applied Analysis of the Gel

### 3.1. Analysis of Gels’ pH

Different types of gels, including transdermal, injectable, and ophthalmic gels, require specific pH ranges to align with their intended pharmaceutical applications and administration routes. As gel formulations are used across various parts of the body, the pH measurement is a crucial factor influencing drug safety and patient comfort. In this section, we present statistical data on the pH ranges of gels targeted for different applications over the past five years, providing a benchmark for future advancements in gel pH optimization.

Ophthalmic gels demand particular attention to pH, with an ideal value of 7.4, corresponding to the pH of tear fluid [121]. Deviations outside the safe range of pH 4–10 can cause ocular irritation and damage. The recent literature indicates that the pH of ophthalmic gels ranges from 6 to 7.63, well within the safety margin, thereby avoiding potential eye irritation [47,122,123,124,125].

For nasal gels, the slightly acidic pH of the nasal mucosa (ranging from 5 to 6.5) can tolerate formulations with pH values between 3 and 10. However, gels with a neutral to slightly acidic pH are optimal for minimizing discomfort, irritation, or harm to the nasal lining while improving mucociliary clearance [126]. Recent studies report pH values for nasal gels between 4.64 and 7.23, conforming to these guidelines [68,127,128,129,130,131].

Transdermal gels require a pH range of 5–7 to prevent skin irritation [132]. Normal skin pH is slightly acidic, ranging from 5.6 to 7.5 [133]. A low pH may reduce drug permeation, while a high pH (above 7) could cause irritation, such as redness or mild burning with prolonged exposure [134]. Recent reports show transdermal gels with pH values ranging from 5.45 to 6.82, which align well with natural skin pH [72,135,136,137,138,139].

The pH of the rectum is approximately 6.8 [140]. Data from recent studies indicate that rectal gels, excluding formulations requiring an alkaline environment for specific drugs, such as pentobarbital, generally maintain a neutral pH range of 6.5–7.4. These values are compatible with rectal mucosa and do not cause irritation or discomfort [141].

For vaginal gels, the pH of a healthy vaginal environment ranges from 3.8 to 5 [142,143]. The literature reports a pH range of 3.7 to 6.7 for vaginal gels, ensuring they remain slightly acidic or neutral, which is both safe and suitable for vaginal application [140,141,144,145,146,147].

The oral mucosa typically has a mean pH of 6.78 [148]. Most oral gels, except those formulated for drugs requiring an acidic environment for solubility, exhibit pH values between 6.8 and 7.4, ensuring compatibility with oral tissues and minimizing inflammation while enhancing patient compliance [149,150,151,152]. Figure 6 summarizes the statistical pH ranges for gels across various administration routes, providing a comprehensive overview of pH requirements tailored to specific applications.

### 3.2. Analysis of Gels’ Structure

The microstructure of gels plays a crucial role in determining their macroscopic properties, making the structural analysis of gels an essential component of their characterization. The evaluation of gel structure typically involves two approaches: microstructure characterization and chemical structure characterization. Since different applications demand varying structural attributes, this section provides a brief overview of gel structure analysis without delving into detailed statistics or results.

Micromorphology is often examined using high-resolution microscopy techniques, such as SEM, TEM, and atomic force microscopy (AFM). These methods consistently reveal that gels possess porous, three-dimensional network structures [71,94,135,150,153,154]. Figure 7 illustrates gel structures captured by different electron microscopy techniques. The porosity and pore size of gels vary based on their intended use. For instance, wound dressing gels are typically more porous and loosely structured to facilitate gaseous exchange of O₂ and CO₂, which is critical for promoting the healing process [155]. Additionally, surface micromorphology often shows a uniform distribution of precursor compounds in gels prepared with precursor formulations.

Chemical structure characterization is vital for confirming the successful encapsulation of drugs and excipients in gel formulations and ensuring that these components do not chemically interact with each other. Techniques such as FT-IR, XRD, and UV spectroscopy are commonly employed, with FT-IR being the most frequently used.

FT-IR spectroscopy is widely applied to evaluate hydrogen bonding, verify gel formation, and confirm drug encapsulation. For example, Eman S. Shalaby and colleagues compared the chemical group spectra of drugs, excipients, and drug-loaded gels to demonstrate that no new characteristic peaks were formed, indicating that the drugs remained in their natural state without chemical interaction with the gel delivery system [67]. The results illustrate FT-IR spectra comparisons, revealing that traditional and optimized organogels did not exhibit new peaks, supporting the absence of chemical interplay. FT-IR spectroscopy can also predict gel cross-linking and propose formation mechanisms. During gel formation, new characteristic peaks may appear in the gel’s spectrum compared to excipient spectra, confirming cross-linking. The precise locations and changes in absorption bands can be analyzed using second derivative transformations of the FT-IR spectra [156]. Table 2 lists the characteristic FT-IR peaks for common gel excipients, including span, tween, carbomer, poloxamer, and chitosan [47,68,69,72,94,157,158].

XRD is another powerful tool for analyzing the crystalline or amorphous nature of gels. The XRD curves of drug-loaded gels often mirror those of the pure drug and excipients, indicating successful encapsulation within the gel’s amorphous network [156]. Furthermore, XRD can demonstrate chemical cross-linking between drugs and excipients, contributing to a more ordered gel structure [159].

While UV and NMR spectroscopy are also utilized in the structural evaluation of gels, their applications are less frequent and, therefore, are not discussed in detail in this section.

### 3.3. Analysis of Gels’ Mechanical Properties

The mechanical properties of gels are crucial evaluation criteria, encompassing gel strength, spreadability, and textural attributes, such as hardness, adhesiveness, and cohesiveness. These properties play a significant role in the efficacy and patient compliance of gel formulations [160]. This section reviews data on mechanical properties from the literature over the past five years, elaborating on testing methods and their physical significance. The testing equipment used to assess gel strength, spreadability, adhesiveness, and cohesiveness is illustrated in Figure 8.

Gel strength is a key parameter reflecting the tensile strength [161] and ease of application and administration of gel formulations [162]. It is determined by the strength of chemical or physical bonds within the gel’s internal structure [163]. The standard method measures the time required for a specific weight (commonly 35 g or 3.5 g) to penetrate a set depth (5 cm or 0.5 cm) into the gel [164,165]. Factors such as temperature, polymer type, and polymer concentration can significantly influence gel strength, affecting bioavailability and drug release rates [166]. The literature reports indicate that suitable gel strength values for nasal gels range from 25 to 50 s [128,130,167,168], while rectal gels are suitable within a range of 10 to 50 s [141,162]. Across different administration routes, most gel strengths are concentrated between 30 and 45 s, providing benefits such as easier filling, better adherence to mucosal surfaces, and simplified product removal from packaging [128,130,141,161,162,163,164,165,166,167,168,169,170,171,172,173,174,175,176,177,178,179,180,181,182,183,184,185,186,187,188,189,190,191,192,193,194,195,196,197,198,199,200,201]. These findings offer valuable benchmarks for gel development and optimization [202].

Spreadability, particularly important for topical formulations, like transdermal gels, impacts patient adherence, application uniformity, dosage transfer, and drug efficacy [203]. Spreadability reflects how easily a gel spreads over the intended application area. A commonly adopted method involves sandwiching the gel between two slides, applying a specific weight on top, and measuring the spreading distance and time. Spreadability is calculated using the formula S = M × L/T, where S is spreadability, M is the applied weight, L is the sliding distance, and T is the time in seconds [69]. An analysis of the literature data reveals that most gels, including transdermal, vaginal, and transungual formulations, have spreadability values ranging from 5 to 25 g·cm/s. This range is considered optimal for ensuring even application, enhancing therapeutic effects, and facilitating product extrusion from packaging [69,138,203,204,205,206,207,208,209,210,211,212,213,214,215,216,217,218,219,220,221,222,223,224,225,226,227].

Textural properties, including adhesiveness and cohesiveness, are essential for assessing gel performance [198]. Adhesiveness reflects the effort required to overcome bonding forces between the gel and an applied probe, measured as the negative area under the force–time curve during retraction [228]. Cohesiveness indicates the internal robustness of the gel structure, measured as the ratio of the area under the second compression cycle to the first compression cycle in a force–time plot (A2/A1).

A review of the literature on texture properties reveals that few studies specify an exact range for appropriate cohesiveness and adhesiveness values, instead providing only raw data. This section analyzes data from various types of gels, including transdermal, vaginal, rectal, and oral formulations, and identifies a common range for adhesiveness values across different routes of administration, which is approximately between 0 and 2.5 mJ [82,83,87,88,142,202,228,229,230,231,232,233,234,235,236,237,238,239,240,241,242,243].

Adhesiveness represents the force required to overcome the bond between the gel and the application surface, such as the skin or mucosa, and reflects the retention time of the drug at the application site. Although no standardized value exists for adhesiveness, most studies indicate that values within this range provide adequate adhesion, making the product more suitable for localized applications and prolonging its effectiveness at the intended site.

Similarly, cohesiveness, which measures the internal structural integrity of the gel and its resistance to rupture, is also frequently reported within the 0 to 2.5 range [82,83,84,85,86,87,88,142,201,233,244,245,246,247,248,249]. This range appears to support optimal gel spreadability and ease of removal from containers, contributing to user convenience and application efficiency. These findings offer a valuable reference for the analysis and optimization of gel texture properties in future product development.

Figure 9 summarizes the values of gel strength, spreadability, adhesiveness, and cohesiveness reported in the recent literature [69,82,83,84,85,86,87,88,128,130,138,141,142,161,162,163,164,165,166,167,168,169,170,171,172,173,174,175,176,177,178,179,180,181,182,183,184,185,186,187,188,189,190,191,192,193,194,195,196,197,198,199,200,201,202,203,204,205,206,207,208,209,210,211,212,213,214,215,216,217,218,219,220,221,222,223,224,225,226,227,228,229,230,231,232,233,234,235,236,237,238,239,240,241,242,243,244,245,246,247,248,249] and statistically organizes these data into box plots. The concentration of these properties within specific intervals highlights their relevance in optimizing gel formulations for various applications, supporting the development of gels with desirable mechanical characteristics.

### 3.4. Application of Rheometers in Gel

A rheometer is a precision instrument designed to measure the rheological properties of materials. It accurately determines deformation and flow behavior under varying conditions and finds widespread application in fields such as material science, chemical engineering, the food industry, pharmaceuticals, petrochemicals, and construction. A rheometer provides critical rheological parameters, including viscosity, elastic modulus, yield stress, and thixotropy, which are essential for material research, production process control, and quality assurance.

The instrument primarily consists of a drive system, measurement system, auxiliary system, and control and data acquisition system. Rheometers are broadly categorized into rotary rheometers and capillary rheometers. Rotary rheometers, which generate basic shear flow through rotational movement, are widely used for studying the rheological properties of liquids and semi-solids. Given their prevalence, this section focuses on rotary rheometers and their working principles, fixtures, and application scenarios for different materials.

Rotary rheometers can be classified based on motor type into stress-controlled and strain-controlled types, with stress-controlled types being more commonly used. The operational principle involves applying specific stress or strain to a sample and measuring its response. Two primary modes of operation are employed: rotational and oscillatory.

To accommodate the diverse measurement needs of various materials, rheometers are equipped with a range of fixtures. These fixtures allow precise analysis of rheological characteristics, aiding researchers and engineers in gaining a deeper understanding of material properties. This understanding contributes to advancements in product development and quality control across numerous industries. Table 3 outlines three common fixtures and their characteristics.

For gels with different applications, the parameters and fixtures used on a rheometer vary. Table 4 provides a list of commonly used rheometer fixtures for gel dosage forms, along with their respective test conditions, offering a valuable resource for selecting the appropriate setup for specific applications.

### 3.5. Analysis of Phase Transition Temperature

The temperature responsiveness of gels is a crucial characteristic that significantly impacts their formation and stability. This property has been harnessed to develop thermoresponsive gels, a class of environmentally responsive systems, by incorporating thermoresponsive polymers. Commonly used polymers in this category include synthetic options, such as poly(N-isopropylacrylamide) (PNIPAAm), poloxamer 188, and poloxamer 407, as well as natural polymers, such as chitosan [104]. The applications of these three polymers are summarized in Table 5.

PNIPAAm is a key thermoresponsive polymer widely employed as a carrier in drug delivery systems. Its historical significance dates back to 1967, when Scarpa et al. first reported its thermal phase transition behavior, characterized by a hydrophobic–hydrophilic phase shift in aqueous solutions upon heating [258]. With a phase transition temperature of 32 °C, PNIPAAm aligns closely with the human body’s surface temperature, making it ideal for medical applications [115]. PNIPAAm’s thermoresponsive nature arises from the equilibrium between its hydrophilic (amide) and hydrophobic (isopropyl) side groups. Below its LCST, it swells by absorbing water, while above this temperature, it shrinks, enabling a sol-to-gel transition [116]. The molecular chains of PNIPAAm consist of hydrophilic amide linkages and hydrophobic isopropyl groups. Below the LCST, hydrogen bonds between water molecules and amide groups stabilize the hydration structure around the isopropyl groups. As the temperature rises, this hydration structure breaks down, and the non-hydrated hydrogen bonds between amide groups, along with hydrophobic interactions from the isopropyl groups, become dominant, causing the water molecules to move away from PNIPAAm (Figure 10) [259]. However, conventional PNIPAAm exhibits slow responsiveness due to dense surface layers formed during the phase transition [260]. To enhance its mechanical strength, adhesiveness, biocompatibility, and drug release properties, PNIPAAm is often blended with excipients like hyaluronic acid (HA), poly (vinyl alcohol) (PVA), and cellulose.

**Figure 11:**
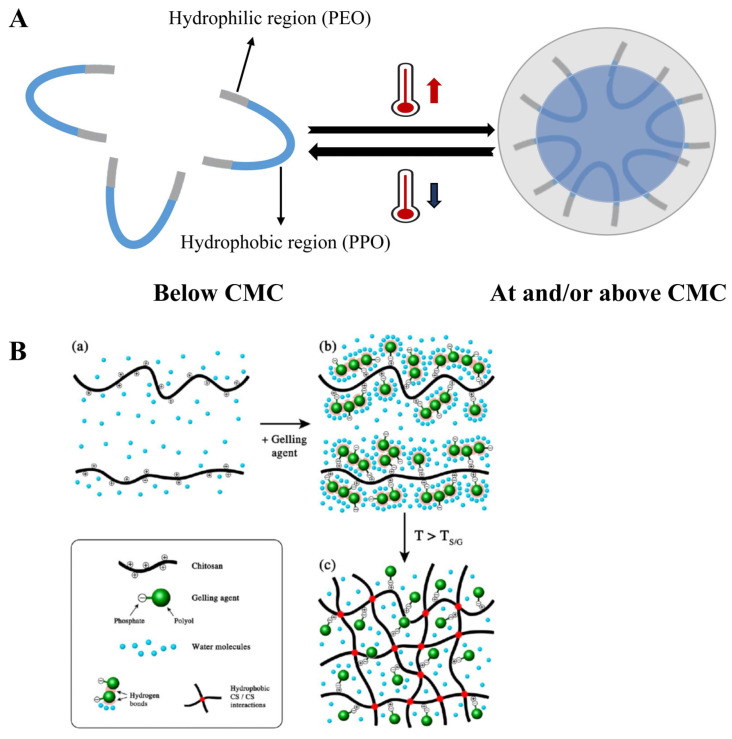
(**A**) The mechanistic diagram of Poloxamer. (**B**) The mechanistic diagram of chitosan. (**a**) initial condition. (**b**) the system begins to change when the gelling agent is added. (**c**) gelatinous condition. Reprinted with permission from [275], copyright 2013, American Chemical Society.

**Figure 1:**
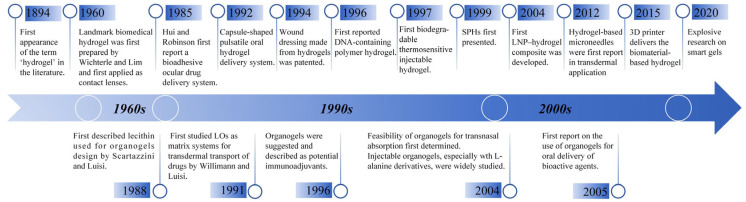
The historical development of gel.

**Figure 2:**
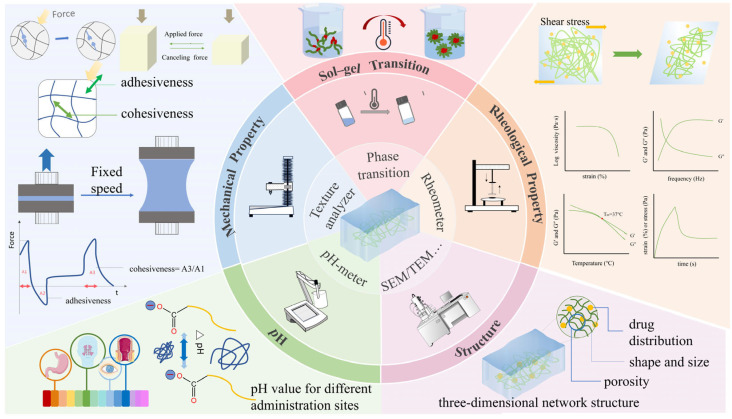
Five common characterizations of gels and their main determination methods mentioned in this review.

**Figure 3:**
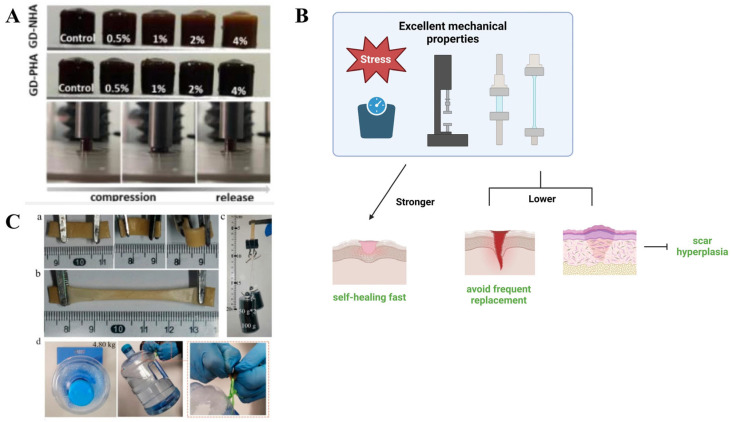
The mechanical properties of the gel applied in different scenarios. (**A**) Image of hydrogel applied in bone tissue engineering during the cyclic compression release test under the texture apparatus. Reprinted with permission from [74], copyright© 2023 Elsevier. (**B**) The advantages of hydrogel applied in wound dressing with good compression resistance. Created with BioRender.com. (**C**) The excellent mechanical properties of gel applied in wearable devices. (**a**) bending, (**b**) stretching, and (**c**,**d**) holding a weight of 200 g and 4.80 kg Adapted from [78].

**Figure 4:**
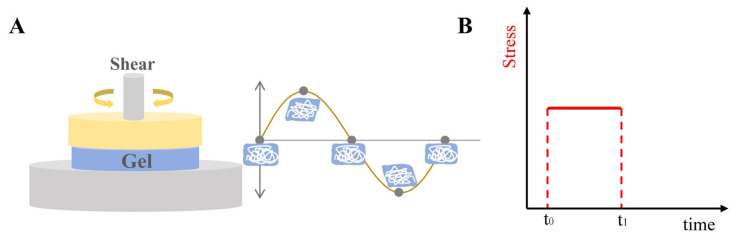
Working principle diagram of rheometer when testing sample. (**A**) Oscillation test; (**B**) creep test.

**Figure 5:**
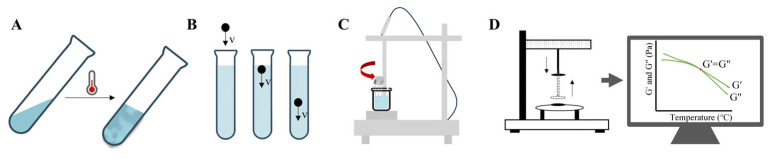
Four methods for measuring the phase transition temperature of thermosensitive gels. (**A**) Test tube inverting method; (**B**) falling ball method; (**C**) magnetic stirrer; (**D**) rotary rheometer.

**Figure 6:**
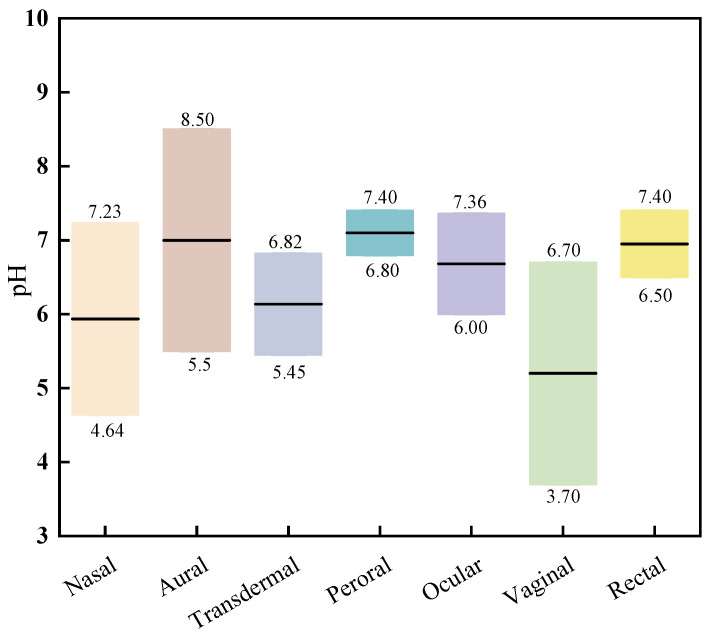
Range of pH values of gels for different routes of administration.

**Figure 7:**
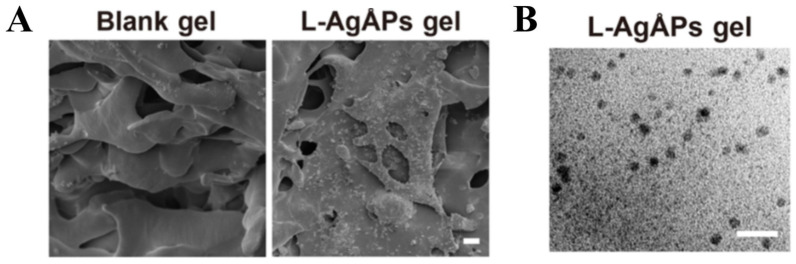
Gel electron microscope structure diagram. (**A**) SEM. (**B**) TEM. Adapted from [94].

**Figure 8:**
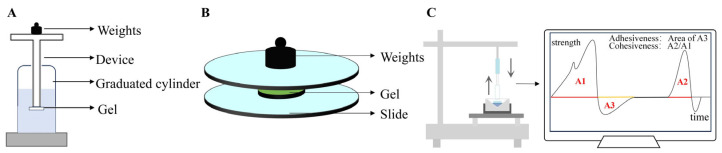
Test equipment used to determine gel’s mechanical properties. (**A**) Gel strength, (**B**) spreadability, (**C**) adhesiveness, and cohesiveness.

**Figure 9:**
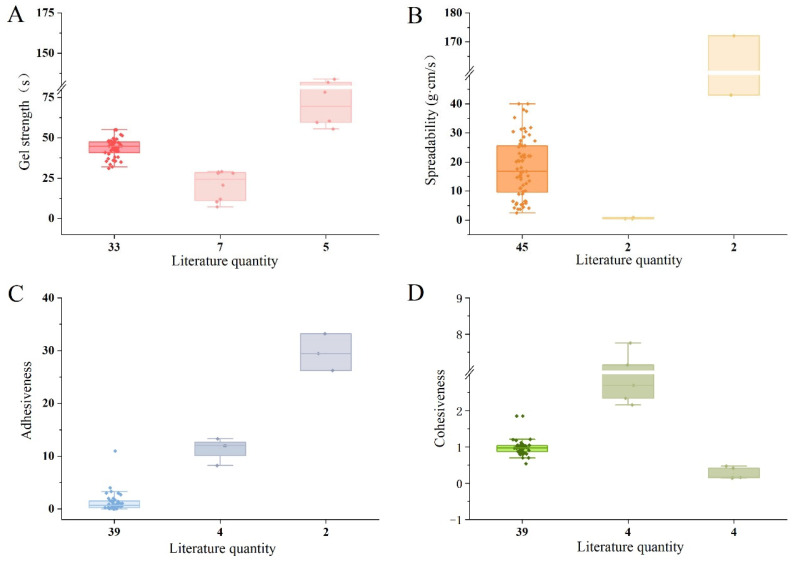
Mechanical property and texture analysis of gels among the literature. Data about (**A**) gel strength, (**B**) spreadability, (**C**) adhesiveness, and (**D**) cohesiveness among the literature in recent years.

**Figure 10:**
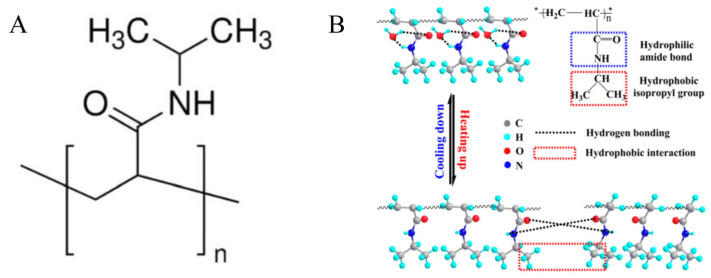
(**A**) The structure of PNIPAAm. (**B**) Thermotropic stretching mechanism of PNIPAAm. Adapted from [259].

Poloxamers, such as poly (ethylene oxide)–poly (propylene oxide)–poly (ethylene oxide) (PEO-PPO-PEO) triblock copolymers, are promising thermoresponsive materials. They consist of hydrophilic polyethylene oxide (PEO) side segments and a hydrophobic polypropylene oxide (PPO) central segment [117]. Poloxamer 407 and Poloxamer 188 are the most commonly used due to their transition temperatures close to body temperature [118]. The PEO/PPO ratio directly influences the sol–gel transition temperature, Tsol-gel, as hydrophobic PPO segments reduce Tsol-gel, while hydrophilic PEO segments increase it [261,262]. By altering the concentration ratio of poloxamers, formulations with suitable Tsol-gel for specific applications can be developed [263]. For example, combining Poloxamer 407, which lowers Tsol-gel, with Poloxamer 188, which raises it, creates formulations with a transition temperature near body temperature, optimizing the gel properties for various drug delivery routes [118]. Poloxamer gelation occurs through micellization, where PPO blocks become dehydrated and aggregate as the temperature rises, leading to sol-to-gel transitions [264,265]. The mechanistic diagram of Poloxamer is shown in Figure 11A.

Chitosan, a natural polymer derived from the deacetylation of chitin, is biocompatible, biodegradable, and safe for biomedical use [266]. It is insoluble in neutral and alkaline pH conditions but dissolves in acidic environments [267]. Chitosan exhibits thermosensitivity due to its LCST of approximately 30 °C, close to body temperature [119]. The introduction of β-glycerophosphate disodium (β-GP) neutralizes the acidic chitosan solution, enhancing hydrogen bonding among chitosan molecular chains. As the temperature increases, hydrogen bonds between chitosan and water break, forming cross-links among chitosan molecules, which results in a gel network [120,268,269,270,271,272,273]. Increasing the concentrations of chitosan and β-GP lowers the sol–gel transition temperature. However, chitosan’s high viscosity, low solubility, and risk of structural collapse without chemical modifications or crosslinking limit its standalone application [274]. To address these limitations, it is often combined with thermosensitive materials, like hydroxypropyl methylcellulose (HPMC) and poloxamers, for improved gel properties and optimal transition temperatures. The mechanistic diagram of chitosan is shown in Figure 11B.

This analysis highlights the versatility of thermoresponsive gels and their suitability for biomedical applications through careful formulation adjustments, paving the way for further advancements in drug delivery and other therapeutic uses.

**Table 5:** Application of common thermoresponsive excipients in gel preparation.

Poly (N-Isopropylacrylamide) (PNIPAAm) with Transition Temperature of 32 °C [115].						
Thermoresponsive Excipients	Other Major Excipients	Purpose of Addition	Application	Tsol-Gel	Reference	Mechanism
PNIPAAm	Hyaluronic acid	Transition temperature of PNIPAAm gels is close to body temperature, so additions used for adjusting Tsol-gel are not necessary. But IPN/semi-IPN network can be formed by adding various hydrophilic polymers to increase mechanical strength, adhesiveness, and biocompatibility and achieve uniform and delayed drug release.	Injection	30~33 °C	[268]	Figure 10B
	Poly (vinyl alcohol)		/	33 °C	[260]	
	Hyaluronic acid		Ocular	34.4~35.5 °C	[276]	
	Hyaluronic acid		Ocular	33 °C	[276]	
	Gelatin		/	37 °C	[277]	
	Salecan		/	32 °C	[116]	
	Cellulose nanocrystals		Wound dressing	36~39 °C	[278]	
Poloxamer with transition temperature of 15~32 °C [279].						
Thermoresponsive excipients	Concentration	Purpose of addition	Application	Tsol-gel	Reference	Mechanism
Poloxamer 407	16.5%	Adjusting concentration of P407 and P188 (P407 usually 15–30% [106]) for the fine-tuning of PPO and PEO ratios to achieve a formulation with an optimal phase transition temperature [280]	Ocular	27.1 °C	[281]	Figure 11A
	15%		Injection	35.3 °C	[282]	
	18%		Ocular	34.3 °C	[283]	
	20.45%		Nasal	31.99 °C	[284]	
	18%		Nasal	32 °C	[285]	
Poloxamer 407/188	20%: 5%		Ocular	28.4 °C	[281]	
	17.3%: 1.2%		Buccal	31.5~ 33.5 °C	[118]	
	24.07%: 1.22%		Rectal	32.8 °C	[265]	
Chitosan with transition temperature of 30 °C [119].						
Thermoresponsive excipients	Other major excipients	Purpose of addition	Application	Tsol-gel	Reference	Mechanism
Chitosan	β-glycerophosphate	Encourage the creation of the gel, enhancing its robustness and the ideal duration for gelation	Injection	37 °C	[286]	Figure 11B
	β-glycerophosphate		Injection	32.6 °C	[269]	
	β-glycerophosphate		Injection	28.36 °C	[270]	
	Gelatin/β-glycerophosphate		Ocular	37 °C	[271]	
	Gelatin/β-glycerol phosphate		/	32.17 °C	[272]	
	Hydroxypropyl methylcellulose/glycerol	Hydroxypropyl methylcellulose aids in thermogelation, while glycerol reduces the temperature of phase transition.	Injection	32 °C	[274]	
Mixed usage of multiple thermoresponsive excipients.						

## 4. Discussion

This document provides a comprehensive overview of the five fundamental properties of gels, along with their corresponding characterization techniques and methodologies. Gels are widely utilized across a range of industries, including food, materials science, chemistry, and physics, with particularly significant applications in biomedical fields. For instance, their rapid and sensitive responses to external stimuli make them ideal for biosensor applications. Additionally, their biodegradability, self-healing capabilities, and antibacterial properties render them suitable for tissue engineering, where they can support the repair of various organs [273]. Furthermore, gel-based stents have demonstrated potential as effective solutions for surgical procedures and implantations due to their flexibility, durability, and biocompatibility, finding use in glaucoma management [287] and ureteral treatment [288]. Gel-based conductive materials have also garnered considerable attention for their stretchability, flexibility, and biocompatibility, enabling significant advances in wearable biomedical devices and healthcare systems [289].

This review focuses specifically on the application of gels in drug delivery systems, including transdermal, oral, ocular, nasal, vaginal, and rectal administration. While numerous studies summarize the applications of gels in pharmaceutical delivery, few reviews explore the detailed characterization methods or the significance of these techniques. Even fewer conduct data analyses of the results. This review aims to fill this gap by highlighting commonly used characterization methods, their importance, and relevant data analyses. The key topics covered include the following: pH characterization—analyzing pH requirements tailored to specific body sites to avoid irritation and ensure safety; structural characterization—examining microstructure and chemical structure using advanced techniques; mechanical and rheological properties—investigating gel strength, texture, and rheology through texture meters and rheometer; and phase transition behavior—assessing thermosensitive gels, their transition principles, and temperature determination.

**Figure 12:**
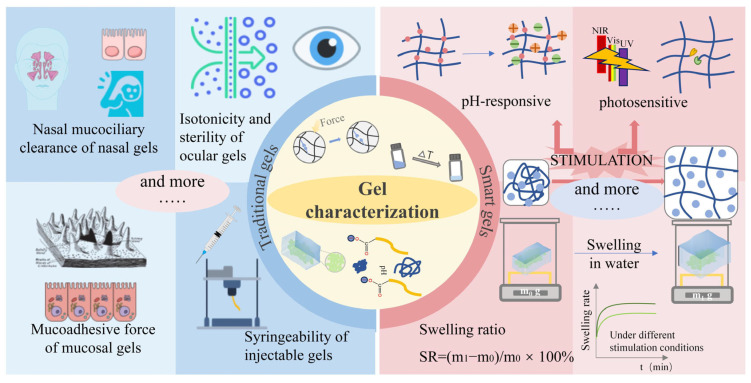
Gel characterizations mentioned in this review and others yet to be supplemented.

The findings presented here provide a valuable reference for researchers developing gel formulations. Figure 12 summarizes the characterization techniques discussed, along with additional potential methods.

Beyond these general characterization methods, gels used for different routes of administration require specialized evaluation techniques and parameters. For example, mucosal gels should be evaluated for mucoadhesive force (e.g., nasal, oral, vaginal, rectal mucosa) [284] and, in the case of nasal gels, mucociliary clearance is often measured [290]; ocular gels are subjected to sterility testing [291], ocular irritation studies [292], and osmotic pressure measurements to maintain isotonicity (290–310 mOsmol/kg) [293]; injectable gels are tested for syringeability to estimate the force required during injection [294]; and general properties, including parameters such as size, polydispersity index (PDI), zeta potential, drug entrapment efficiency (%EE), visual appearance, and clarity, are assessed, particularly in gels incorporating nanoparticles or lipogels as drug carriers [242].

While the five key characterizations outlined are general to traditional gels, phase transition temperature is a unique feature of thermosensitive gels. Additionally, environmentally sensitive gels, including photo-, electro-, magnetic-, pH-, ion-, and enzyme-sensitive gels, represent a burgeoning area of research. These gels require distinct characterization techniques tailored to their specific principles. For instance, swelling behavior in media with varying pH levels or light conditions serves as a primary evaluation metric for pH-sensitive and photo-sensitive gels [295,296]. Given the growing interest in environmentally responsive gels, further research is needed to develop exclusive characterization methods beyond the five commonly discussed in this review.

In recent years, 3D-printed hydrogels have been extensively studied due to their excellent biocompatibility [297], ability to release drugs on demand [298], and other advantageous properties. Therefore, 3D-printed hydrogels can be used in bioengineering applications, such as wound healing [299], tissue engineering [300], and cancer treatment [298,301,302,303,304]. The common structural features of 3D-printed hydrogels include porous structures [305], lattice structures [306], and hierarchical structures [307]. Since this article primarily focuses on the synthesis and characterization of gels, the principles and application pathways of 3D-printed hydrogels are not described in detail. Additionally, the use of hydrogels for cancer therapy has become a prominent area of research, particularly in their application as drug delivery systems for treating breast cancer [297,308,309], localized cervical cancer [310,311], and lung cancer [312], among others.

Gels are highly versatile materials that can be tailored through simple formulation adjustments to create structured systems with significant potential in drug delivery. However, several challenges remain. For example, the biocompatibility of organogels compared to other gel systems has not been thoroughly investigated. Future research should prioritize designing bio-based organogels with biocompatible solvents, addressing matrix degradation, mitigating by-product effects, and enhancing the applicability of gels for advanced drug delivery systems. Continued innovation in gel formulations and characterization methods will be essential to unlocking their full potential in biomedical applications.

## Figures and Tables

**Table 1:** Rheological tests and their parameters, images, and significance.

Test	Measurement Methods	Image	Common Rules	Significance
Strain sweep	Characterize gels through the application of escalating oscillatory strain at a steady frequency	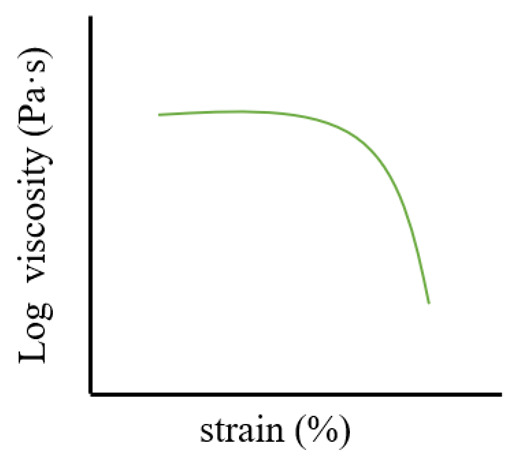	In a specific area of gels experiencing low shear stress, the moduli remain unaffected by the escalating stress.	To determine LVR region of gels
Frequency sweep	Assess the viscoelastic characteristics of gels through a comparison of G′ and G″ values across various frequencies	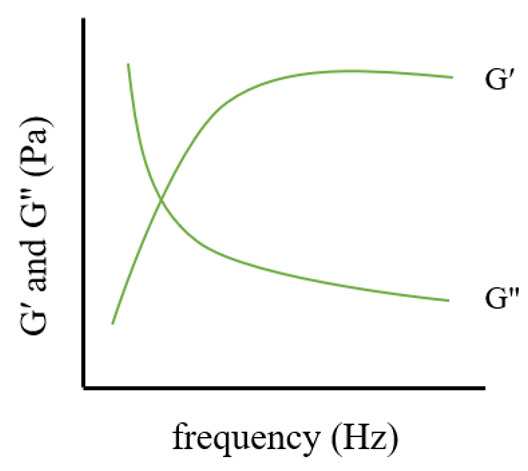	Every gel exhibits characteristics of a viscoelastic fluid, characterized by upward gradients on the G′ and G″ axes, leading to a more rapid rise in the loss modulus.	To determine the G′ and G″ crossover points
Temperature sweep	Measure transition temperature of gels by increasing temperature and record when G′ value equals with G″ value.	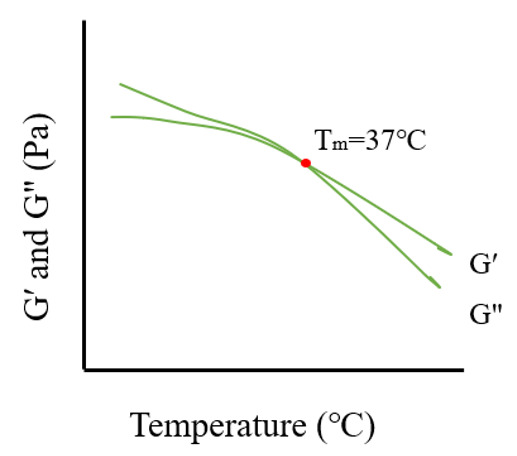	As temperature increases, G′ and G″ show a downward trend, and there will exist a crossover point at a certain temperature, known as the phase transition temperature.	To determine phase transition temperature; especially used in thermosensitive gels
Creep test	Record compliance over time when applying stress and after stress relief	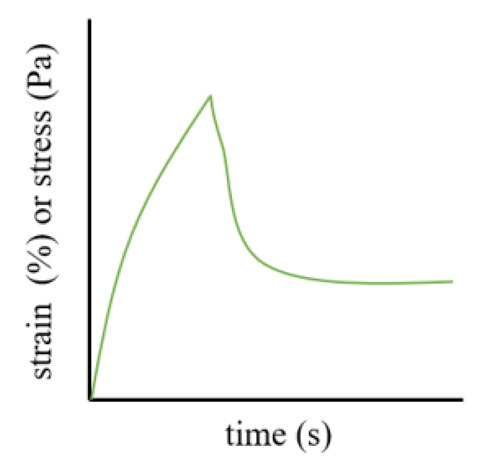	During the loading step, the J value is inversely proportional to the stiffness and recovery time, and the slope and creep compliance during the recovery step can reflect the elastic behavior and stiffness of the gel.	To evaluate the stiffness and elasticity of gels

**Table 2:** Gel structure characterization methods.

Structural Characterization	Methods	Aim	Data Analysis	Reference
Microstructure Characterization	SEM/TEM/AFM, etc.	To observe micromorphology of gel	Shape or size	[71,94,135,150,153,154]
	FT-IR	The aim is to determine the potential interplay between excipients and active drugs in gel formulations.	Span60 (-CH) 2956 and 2849 cm^−1^ (-OH in carboxyl group) 2916 cm^−1^ (-C=O in ester) 1736 cm^−1^ (-CH) 2930, 2899, 2867, and 2834 cm^−1^ (-C=C in alkenes) 1671 cm^−1^	[158]
			Span20 (-OH) 3392 cm^−1^ (-CH2- asymmetric stretching) 2923 cm^−1^ (-CH2-symmetric stretching) 2834 cm^−1^ (-C=O stretching) 1738 cm^−1^	[157]
			Tween20 (-OH stretching) 3488 cm^−1^ (-CH2-asymmetric stretching) 2920 cm^−1^ (-CH2-symmetric stretching) 2860 cm^−1^ (-C=O stretching) 1734 cm^−1^	[157]
			Carbomer (-NH and -C=O) 3357 cm−1 and 1639 cm^−1^	[94]
			Carbomer with the existence of triethanolamine (-N-H) around 3316 cm^−1^ (-C-H) 2931 cm^−1^ (-C-O-C) 1200 ~1250 cm^−1^ (=C-H) 800~850 cm^−1^ 1643 cm^−1^ peaks signify the presence of hydrogen bonds in the carbonyl group of carbopol when hydrated.	[72]
			Poloxamer 407 (-C-O-C) 1240 cm^−1^ (-O-H) 3647.2 cm^−1^ (-N-H) 3347 cm^−1^	[68]
			Chitosan (-O-H and -N-H) 3348 cm^−1^ (-C-H) 2867 cm^−1^ (-CONH2) 1643 cm^−1^ (-C-O) 1072 cm^−1^	[69]
			Sodium alginate (mannuronic acid) 880 cm^−1^ (uronic acid) 1056 cm^−1^ (-OH) 2283 cm^−1^ (-CH2) 2928 cm^−1^	[47]

**Table 3:** Types and characterizations of rheometer fixtures.

Types	Pattern	Composition	Peculiarity	Range of Application	Notes
Coaxial cylinder fixture	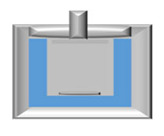	It consists of an inner cylinder and an outer cylinder. The inner cylinder can rotate, while the outer cylinder remains stationary. The sample fills the annular space between the inner and outer cylinders.	The diameter of the inner cylinder is lesser, whereas the outer cylinder exhibits a greater diameter, and both are approximately equal in height.	1. Low-to-medium viscosity fluids 2. Some organic solvents or easily oxidized liquid samples	Ensure the inner and outer cylinders are properly aligned during their setup and operation.
Taper plate fixture	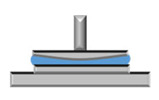	It consists of a cone and a flat plate. The apex of the cone is in close contact with the plate, and the sample fills the small space between the cone and the plate.	The angle of the cone is usually small, typically between 1° and 4°, to ensure relatively uniform shear stresses and shear rates during the measurement.	1. High-viscosity fluids, as well as pasty and semi-solid samples 2. Precious samples	1. The cone and plate need to be carefully cleaned to avoid the impact of residual samples on subsequent measurements. 2. Pay attention to temperature control.
Parallel plate fixture	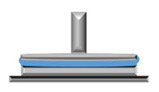	It consists of two parallel disks. The sample is placed between the two disks, the diameter of which can be selected according to the amount of sample and the measurement requirements.	The spacing between the two disks can be precisely adjusted by the instrument to accommodate samples of different viscosities and thicknesses.	The fluids of various viscosities, as well as thin film-like and sheet-like samples	1. Ensure the sample is evenly distributed between the parallel plates. 2. Prevent sample edge extrusion. 3. Pay attention to the temperature uniformity between the parallel plates.

**Table 4:** Rheometer test methods for selected dosage forms.

Gel Type	Rheometer Model	Fixture	Temperature/°C	Shear Rates/s^−1^	Frequency	Strain/%	Reference
Oral gel	/	/	25 ± 1	2, 10, 20, 30, 40, 50, 60	/	/	[250]
	/	/	25 ± 5	2, 10, 20, 30, 40, 50, 60	/	/	[251]
	ARES-G2, TA	steel cone	15~45	shear stress (0.1~500 Pa)	/	0.1	[252]
Gel injection	Anton Paar Physica MCR 300	cone plate	25	0.7 Hz	0.7 Hz	0.1	[253]
	Anton Paar Physica MCR 300	cone plate	20	6.28 rad	0.0628 Hz~62.8 rad/s	0.01~10	[253]
	Kinexus Pro, Malvern	parallel plate	25~45	/	0.1~100 Hz	1	[254]
Topical gels	TA	parallel plate	25	0.1~200	0.1~100 rad/s	5	[255]
	TA, AR-500	cone plate	/	/	1~100 rad/s	/	[256]
	MCR502, Anton Paar	parallel plate	/	/	1 Hz	0.001~10	[257]
Ophthalmic gel	/	parallel plate	25 ± 0.1	/	angular velocity (0.5~100 rpm)	/	[122]
	Anton Paar, MCR 302	parallel plate	20~37	/	/	/	[123]
	/	/	34 ± 1	/	angular velocity (0.5~100 rpm)	/	[124]
	/	vaned rotor	25	1000~10^−5^	/	/	[125]

## Data Availability

Not applicable.
